# Intercellular Communication during Stomatal Development with a Focus on the Role of Symplastic Connection

**DOI:** 10.3390/ijms24032593

**Published:** 2023-01-30

**Authors:** Yongqi Cui, Meiqing He, Datong Liu, Jinxin Liu, Jie Liu, Dawei Yan

**Affiliations:** 1 State Key Laboratory of Crop Stress Adaptation and Improvement, School of Life Sciences, Henan University, Kaifeng 475001, China; 2 Key Laboratory of Wheat Biology and Genetic Improvement for Low & Middle Yangtze Valley, Ministry of Agriculture and Rural Affairs/Lixiahe Institute of Agricultural Sciences of Jiangsu, Yangzhou 225007, China; 3 Academy for Advanced Interdisciplinary Studies, Henan University, Kaifeng 475001, China

**Keywords:** stomata, plasmodesmata, guard cell, subsidiary cell, symplastic communication

## Abstract

Stomata are microscopic pores on the plant epidermis that serve as a major passage for the gas and water exchange between a plant and the atmosphere. The formation of stomata requires a series of cell division and cell-fate transitions and some key regulators including transcription factors and peptides. Monocots have different stomatal patterning and a specific subsidiary cell formation process compared with dicots. Cell-to-cell symplastic trafficking mediated by plasmodesmata (PD) allows molecules including proteins, RNAs and hormones to function in neighboring cells by moving through the channels. During stomatal developmental process, the intercellular communication between stomata complex and adjacent epidermal cells are finely controlled at different stages. Thus, the stomata cells are isolated or connected with others to facilitate their formation or movement. In the review, we summarize the main regulation mechanism underlying stomata development in both dicots and monocots and especially the specific regulation of subsidiary cell formation in monocots. We aim to highlight the important role of symplastic connection modulation during stomata development, including the status of PD presence at different cell–cell interfaces and the function of relevant mobile factors in both dicots and monocots.

## 1. Introduction

Stomata are cell complexes on the plant epidermis for gas and water exchange between plants and environment [1]. The dynamic change of the pore size controls the loss of water and promotes the absorption of carbon dioxide in plants, enabling them to function as essential regulators of photosynthesis and transpiration [2], as well as growth and development [3,4]. Stomata are also one of the channels for pathogens to invade plants [5], and plays an important role in plant responses to abiotic and biotic stresses [6,7,8,9,10].

The stomata of dicot are usually composed of a pair of kidney-shaped guard cells that are produced through a series of asymmetric and symmetric cell divisions, which also modulate the overall density and pattern of stomata [11]. In most dicot leaves, stomata appear to be randomly distributed on the epidermis of dicotyledons but with ‘one-cell spacing rule’, in which two stomata are separated by at least one intervening nonstomatal epidermal cell [12]. In contrast, the stomata of monocot are kidney- or dumbbell-shaped and arranged in parallel with uniform distribution [13,14,15]. In addition, a significant feature of stomata in monocot is the presence of one or more pairs of subsidiary cells flanking the guard cells that are different in form, size or arrangement compared with regular epidermal pavement cells [16]. Subsidiary cells (SCs) provide mechanical support or act as a reservoir for water and ions supporting the movement and function of guard cells (GCs) [17].

Apoplast and symplast transport are two ways of intercellular communication in plants [11,18,19]. In the apoplast pathway, molecules are transported from the extracellular matrix to the cytoplasm, or as ligands to target receptors in the extracellular layer. In contrast, the symplast pathway is carried out through nano channels, named plasmodesmata (PD) [20,21,22]. Symplastic communication via PD are essential for a wide range of plant biological activities, including organ formation, tissue patterning and stress responses [23,24,25,26,27,28]. PD are channels composed of plasma membrane (PM) and endoplasmic reticulum (ER), which provides cytoplasmic and membrane continuity between adjacent cells for the trafficking of metabolites and signaling molecules [27,29,30,31]. Defects in PD structure and connectivity limit the intercellular transport of sugars, ions, transcription factors and a series of RNA molecules including messenger RNA (mRNAs), short interfering RNA (siRNA), microRNA (miRNAs), trans acting siRNAs (tasiRNAs), and also the hormone distribution [32,33,34,35,36,37].

Based on the inner structure, PD are classified into two types, simple and branched (X-, Y- and V-shaped) [38]. According to the presence or absence of cytoplasmic sleeve, it can be divided into type I (no) and type II (yes). Simple PD have higher permeability than branched PD in mature leaves, while type I PD exhibit higher permeability than type II PD in young tissues [39]. At present, many relative studies focusing on the plasmodesmal structure and components have been reported [40,41,42]. The PD aperture is mediated by the change of flanking cell walls, mainly by the turnover of synthesis/degradation of callose. Callose is a β-1,3-linked homopolymer of glucose with a minority of β-1,6-branches synthesized with UDP-glucose as substrate [43]. Callose levels are regulated by two antagonistic enzymes: callose synthase (CalS) or glucan synthase-like (GSL) for *Arabidopsis* only and β-1,3-glucanases, which produce and break down callose, respectively [44,45].

In this review, we briefly summarize the conserved and distinct regulation mechanisms underlying stomata development in dicots and monocots and the specific regulation of SCs formation in monocots. Then, we focus on the comparison of the modulation of symplastic communication during stomatal development in both dicots and monocots based on recent studies. Although there are not too many cases in this field at present, our review would be of help for further deep investigation. 

## 2. A Brief Summary of Key Transcription Factors Regulating Stomatal Development

**Figure 1:**
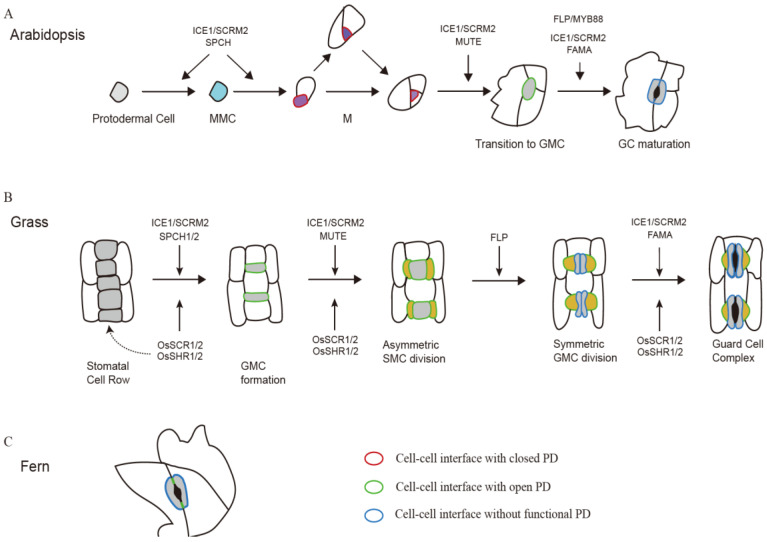
Schematic diagrams showing key regulators and symplastic connections during stomatal development in *Arabidopsis*, Grass and Fern. (**A**) Stomatal development in *Arabidopsis*. A protodermal cell converts to the stomatal lineage when it becomes a meristemoid mother cell MMC, then the MMC undergoes an asymmetric division to produce a smaller meristemoid (purple) and a larger stomatal lineage ground cell (SLGC; white). The meristemoid (M) undergoes additional self-renewing asymmetric divisions or differentiates into a guard mother cell (GMC; light gray). The GMC divides symmetrically to form a pair of guard cells (GCs; gray). Five bHLH transcription factors, SPCH, MUTE, FAMA and their heterodimeric partners ICE1 and SCRM2 regulate fate transitions during stomatal development. SPCH and ICE1/SCREAM2 mediate both entry and [54] amplifying asymmetric divisions, while MUTE and ICE1/SCREAM2 mediate the transition from M to GMC fate. FAMA, FOUR LIPS (FLP)/MYB88, and ICE1/SCREAM2 control the GMC symmetric division and GC maturation. Plasmodesmata (PD) were observed in ventral walls between sister guard cells and, most importantly, between immature GCs and epidermal cells, but degenerated until GC became mature. However, the PD are supposed to be closed to block the intercellular movement of transcription factor SPCH before GCs formed. (**B**) Stomatal development in grasses. The developmental process of stomata in grasses can be divided into five steps: (1) specification of the stomatal file, (2) GMC formation, (3) asymmetric division of subsidiary mother cells (SMCs) to generate SCs, (4) GMC symmetric division to form two immature GCs, (5) maturation of two dumbbell-shaped GCs flanked by two triangle-shaped SCs. *SPCH* has been duplicated and both homologues act to induce asymmetric divisions and promote the acquisition of stomatal lineage identity, in which *SPCH2* plays a predominant role. The grass MUTE has a unique character to move from GMCs into SMC where it functions to promote the asymmetric division of SMCs to form SCs. FAMA and FLP regulate the symmetric division of GMCs and the maturation of GCs. Like the *Arabidopsis* counterparts, grass ICE1/2 proteins form heterodimers with SPCH1/2, MUTE, or FAMA, respectively. In rice, SCARECROW (SCR)1/2 and SHORTROOT (SHR)1/2, have been shown to act together to regulate the initiation of stomatal lineage cells, formation of SCs, and maturation of GCs. Before the complete maturation of stomata complex, there are functional PD connecting GMCs with surrounding cells. However, PD are just present between ordinary epidermal cells and SCs when mature GCs formation, but not GC-GC and GC-SC interfaces. (**C**) Stomata of fern. A few long and narrow PD are present between two GC cells in fern, allowing the fluorescent dyes slowly move from guard cell to another, but the electrical conductance of the PD is not high enough to provoke membrane potential changes.

The orderly development of stomatal complex is regulated by many pathways, including a series of transcription factors, signal peptides and mitogen-activated protein kinase (MAPK) signal pathway [46]. Among them, basic Helix-Loop-Helix (bHLH) family transcription factors play an important role. SPEECHLESS (SPCH), MUTE and FAMA belong to the same subgroup showing certain similar structure but different function [47]; INDUCER OF COLD EXPRESSION(ICE1)/SCREAM (SCRM) and SCRM2 have functional redundancy (Figure 1). Experiments showed that they function with SPCH, MUTE and FAMA in heterodimers with a dose effect [47]. SPCH acts on the initial stage of stomatal cell formation, mainly affecting the transformation from protodermal cell (PDCs) to meristemoid mother cells (MMCs), which undergo self-renewal and differentiate to meristemoids. The *SPCH* mutation in *Arabidopsis* significantly reduced the stomatal index, whereas overexpression of *SPCH* induces extra cell divisions in epidermal cells that do not normally divide [48]. Recent studies have revealed the signal molecules *SOL1* and *SOL2* are the transcriptional targets of SPCH and they function as negative regulators on fate transitions of the stomatal lineage, and these two genes are enriched in stomatal precursors. *TSO1*, a paralog of *SOL1* and *SOL2*, acts in opposition to them during this process [49]. AtMUTE is required for the transition from M to guard mother cell (GMC), it also ensure the symmetrical division of GMC into GC by inducing cyclin (CYCD5; 1) and other cell cycle factors [50]. Loss-of-function mutations of *MUTE* lead to the developmental progress getting stuck in a continuous self-renewing stage [51]. Almost all epidermal cells were converted into stomatal precursors in *35S::MUTE* plants [14]. FAMA mediates the symmetrical division of GMC to generate GC. FAMA is only expressed in GMCs and early GCs. GMC division in the *atfama* mutant is blocked and GMCs cannot differentiate into GCs [3]. The overexpression of *FAMA* will induce other mesophyll cells to gain the stomata fate. In addition, FAMA directly inhibit *CYCD7; 1* to prevent excessive division of GCs [52]. Two MYB transcription factors FLP and MYB88 have functional redundancy with FAMA, which are responsible for GMC division regulation. The stomatal phenotype of the double mutant *flp myb88* is consistent with the *fama* mutant [53].

Compared with *Arabidopsis*, some transcription factors of monocots that play key roles in stomatal development are somewhat different in functions. According to previous studies, three grass species-maize, rice and *Brachypodium distachyon*-have only one orthologue of *Arabidopsis MUTE* and *FAMA*, but two of *SPCH* [55]. The protein sequences of these transcription factor genes are highly conserved between these two typical angiosperms. Lots of functional differences of these TFs between grass and *Arabidopsis* have been well studied. The AtSPCH protein is highly regulated by phosphorylation, but the phosphorylation regulatory domain is not conserved in BdSPCH1/2. Overexpression of *AtSPCH* induced excessive epidermal cell divisions, and *35S::OsSPCH1* showed normal stomata phenotype, but *35S::OsSPCH2* can promote cell division in the pavement cells [48,56]. The *bdspch1* single-mutants showed a 20% reduction in stomatal numbers, while the loss of *BdSPCH2* would lead to an 80% reduction [57]. *SPCH1/2* of grass plays a role in promoting stomatal development, and *OsSPCH2* and *BdSPCH2* play a more important role during this process than *OsSPCH1* and *BdSPCH1*, respectively. The function of grass *SPCH* is not completely equivalent to *AtSPCH*. Grass *MUTE* regulates stomatal development but in a divergent way to *AtMUTE*. MUTE in *Arabidopsis* control the cell fate transition from M to GMC, and from GMC to paired GCs [50]. BdMUTE appears to function in the recruitment and establishment of SC identity, and it expresses not only in GMCs and subsidiary mother cells (SMCs), but also in young GCs and SCs. Moreover, it can travel to and accumulates in the adjacent neighbor cells through PD [58]. OsMUTE is also mobile but it is induced restrictively in GMCs and SMCs, and it controls GC shape by positively regulating *OsFAMA* expression [59]. Loss of *OsMUTE* or *ZmMUTE* led to more severe consequences for GMC identity compared with mutant of *BdMUTE* [59]. The loss-of-function mutants of *ZmMUTE* failed to recruit SCs and resulted in longitudinal division of GMC [60]. However, the loss-of-function mutants of *BdMUTE* showed almost 70% normal GMC division, which is totally different from loss-of-function mutants of *OsMUTE* and *ZmMUTE* that completely lack stomata [58]. Overexpression of *AtMUTE* and *ZmMUTE* in *Arabidopsis* led to other epidermal cells developing into stomata [56], but *Ubip:YFP-BdMUTE* showed the stomata phenotype such as *35S::SPCH* [59]. These differences in the role of MUTE might be due to variances in the protein structure or interacting partners. OsFAMA controls the cell fate transition of GMCs and SMCs, but not cell division that AtFAMA does in *Arabidopsis* [55]. Ectopic *AtFAMA* expression induced the conversion of epidermal cells into GC-like cells, but overexpression of *OsFAMA* usually resulted in the formation of box-shaped GCs because OsFAMA only regulates GC differentiation without the function of GC morphogenesis. *osfama* showed stomata lacking one SC, which indicates OsFAMA contributes to the recruitments of SCs [55,59]. When transforming *ProFAMA:OsFAMA* into *osfama*, it can only restore the phenotype of GCs differentiation rather than the cluster stomata [59,61].These evidence suggest that the function of FAMA is relatively conserved between monocots and dicots, and the grass FAMAs probably have lost their ability to regulate GMC fate. In contrast, the roles of *MUTE* and *SPCH* are discrepant to some extent.

In addition to the previously mentioned more ordered arrangement of stomata in monocots, a pair of dumbbell-shaped GCs flanked by two triangular SCs is another key character. The dynamic open and closure of GC are engaged by ions (K^+^, Cl^−^), which are absorbed and stored in SCs when stomata are closed and transported back to GCs until stomata open. SCs are thus acting as dynamic reservoirs of K^+^ and Cl^−^, facilitating quick and efficient modulation of stomatal aperture by GCs to control water loss. Many experiments indicate that SCs play a key role in enhancing drought tolerance. SCs are formed by an asymmetric division, with cell polarization. The cell polarization is controlled by the BRICK (BRK)–PANGLOSS (PAN)–RHO FAMILY GTPASE (ROP) pathway and other intrinsic or extrinsic cues [62]. This pathway consists of several plasma membrane-associated proteins, all of them are polarized within SMCs. BRK1 belongs SCAR/WAVE regulatory complex, which activates the actin-nucleating ARP2/3 complex and is required for PAN polarization, further promotes branched actin networks [63,64]. PAN1 and PAN2 are two catalytically inactive leucine-rich repeat receptor-like kinases (LRRKs) [65]. PAN2 acts upstream because it is required for the polarized accumulation of PAN1 and PAN2 interacts with itself for its own localization [66]. Type I Rho family GTPases (ROPs) 2 and 9 function together with PAN1 in the polarization of asymmetric SMC divisions. The polarized localization of ROPs depends on PAN1, loss of ROP2/9 causes abnormal SMC division polarity. When these proteins are polarized accumulation at the GMC/SMC contact sites, then the actin patch will form and the nucleus of SMC will migrate toward the GMC [56]. The actin filaments in SMC may provide tracks for polarized organelle movement or facilitate endo/exocytosis at the GMC/SMC interface [67,68,69]. *PAN1* and *PAN2* are two downstream targets of BZU2/ZmMUTE as ZmMUTE directly binds to the promoters of *PAN1* and *PAN2* [60]. 

## 3. Peptide Signaling and Environmental Factors in Stomatal Development

In *Arabidopsis*, two stomata are separated by at least one intervening epidermal cell when they are produced, known as the “one cell spacing rule”, which guarantees the suitable open and closure of stomata [70]. This process is performed by small peptides, ligand receptors, and MAPKs. Secreted small peptides encoded by epidermal patterning factor-like (EPFL) family genes including *EPF1*, *EPF2*, *STOMAGEN/EPFL9* and *CHALLAH (chal)*/*EPFL6* signal inhibit or promote stomatal development in a way of intercellular communication [71,72,73]. *EPF1* and *EPF2* are specifically expressed in the stomatal lineage cells. They are perceived by a receptor complex consisting of a receptor-like protein, TOO MANY MOUTHS (TMM), and ERECTA family (ERf) receptor kinases, followed by the activation of a MAPK module [74]. The MAPK phosphorylated and destabilized the transcription factor SPCH, ultimately led to a reduction in stomatal number and density [75]. The “one cell spacing rule” was broken in *epf1* and *epf2* mutants, which showed clustered stomata and appeared similar to the *SPCH* overexpression plants [72]. Protein phosphatase 2A(PP2A) functioned as a upstream regulator of SPCH by promoting its protein stability, whereas SPCH can directly binds to the PP2A-A subunits in vitro [76]. *EPFL9* is a positive regulator of stomatal development, which is coexpressed with *EPF1* and *STOMATAL DENSITY AND DISTRIBUTION 1* (*SDD1*) [77]. EPFL9 acted independently of EPF1, EPF2 and SDD1, and EPFL9 competes with EPF1 and EPF2 for binding to the ERf-TMM complex [78,79]. EPFL6, expressed only in hypocotyls and stems, was able to suppress stomatal formation and functioned as a suppressor of the TMM [80]. Exogenous application of bioactive BdEPF2 also inhibited stomata initiation, and meanwhile antagonized with BdSTOMAGEN [81]. These results indicate EPF peptides have relatively conserved function in different species. In addition, the CLE peptide family also regulates stomatal patterning. CLAVATA3/ESR-RELATED 9/10 (CLE9/10) functions differently in different plant tissues. CLE9/10, negatively regulates the division of MMCs in leaves, were recognized by the receptor kinase HAESA-LIKE1 (HSL1) and result in the phosphorylation and destabilization of SPCH [82]. 

Apart from the key genetic regulators, environmental factors, including light, carbon dioxide (CO_2_) levels and pathogen attack, as well as endogenous signals, such as abscisic acid (ABA) and apoplastic reactive oxygen species (ROS) also affect the development and movement of stomata [83,84]. Dark-grown wild-type *Arabidopsis* plants showed only a few mature stomata on the cotyledons. Many of them are retained in the stomatal precursor stage or form stomatal clusters occasionally [85,86]. In *Arabidopsis*, the expression of *STOMAGEN*, *SPCH*, *MUTE*, *FAMA*, *EPF2*, and *TMM* can be induced by light [87], and the maturation of stomata complex is disrupted in some photoreceptor mutants such as *phyB*, *phyA*, and *cry1cry2* [88]. In rice, the developed leaves of *phyB1* or *phyB2* mutant displayed larger epidermal cells than wild-type leaves, resulting in the reduction in stomatal density and smaller stomata [89]. The expression of *ZmPIF1* in rice-enhanced tolerance to drought stress, because it plays an important role in the ABA-mediated regulation of stomatal closure [90]. These studies suggest the light-signaling components between grasses and *Arabidopsis* have relatively conserved function. CO_2_ functions as a main substrate for photosynthesis, which can be uptaken into the plant body via stomata. CO_2_ can also meditate both stomatal development and movements. In the *Arabidopsis* GCs, CO_2_ can be catalyzed to HCO_3_^–^ and protons by two carbonic anhydrases of *βCA1* and *βCA4* [91]. In the grass, disruption of *ZmβCA1*, *ZmβCA2* and *OsβCA1* led to an increase in stomatal conductance and decreased sensitivity to high CO_2_ concentrations [92,93]. However, impaired stomatal development in *Arabidopsis βca1 βca4* double mutants was not found in grasses [91].

Phytohormones function in regulating not only various aspects of development and plant growth, but also stomatal patterning. Exogenous ABA application repressed the entrance of stomatal-lineage development rather than the asymmetric division [94]. Disruption of ABA biosynthesis led to disturbed stomatal numbers [95]. The overexpression of *ZmWRKY79* in *Arabidopsis* led to enhanced drought tolerance by inducing the expression of ABA biosynthetic related genes to promote stomatal closure and lateral roots growth [96]. Exogenous treatment with methyljasmonate (MeJA) negatively regulates stomatal development in the cotyledon of *Arabidopsis* [97]. JA-biosynthetic mutants (*jar1*) and signaling-related mutants (*myc2myc3myc4*) exhibited increased stomatal clusters in *Arabidopsis* cotyledons. MYC2/3/4, the key components of JA signal pathway, suppressed the expression of *SPCH*, *MUTE*, and *FAMA* [98]. In rice, *OsJAZ9* regulated potassium homeostasis and stomatal density to enhance the water-deficit stress, and the overexpression of *OsJAZ9* led to the enhanced JA level and reduced stomata density [99].

Long-distance signaling also plays an important role in the adaptation to severe environmental conditions of plants. CLAVATA3/EMBRYO-SURROUNDING REGION related25 (CLE25) peptide is an example of long-distance signaling molecule modulating stomatal closure. It was synthesized in roots under water-deficient condition and transported via vasculature to leaves, and then perceived by the receptors BARELY ANY MERISTEM1 (BAM1) and BAM3 to regulate ABA synthesis and stomatal aperture eventually [100].

Callose accumulation is spatiotemporally participated in numerous plant developmental processes and environmental stress responses, such as cold [101], heat and heavy metals [102,103]. Callose is deposited in the cell wall, PD and sieve pores and can prevent the further diffusion of the pathogen and metals into the plant tissue, and maintain the homeostasis of cellular water [104]. Previous studies showed that limited synthesis or degradation reduced the sensitivity of stomata to light in the fern *Asplenium nidus* [105]. In the young stomata of angiosperms, callose appears in the young ventral wall and might act on the formation of stomata. However, in the mature stomata complex of angiosperm, callose seems to be implicated in stomatal movement [106]. To date, direct evidence showing stress-induced excess callose production directly affects stomatal formation or function is still lacking.

## 4. Symplastic Communication during Stomatal Development

Multiple studies over the past few years have underscored the crucial role of PD in plant development by governing movement of biological macromolecules that determine cell fate. Some regulators of stomatal formation possessing the ability to traffic through PD have been revealed (Table 1). miRNAs play a key role in stomatal development, miRNA-deficient mutants, such as *dcl1*, *hen1* and *ago1*, display abnormal in stomatal density and patterning. The MADS box gene AGAMOUS-LIKE16 (AGL16), expressed in GCs, could be cleavage by *miR824*, which is expressed in GMCs, meristemoids and stomatal-lineage ground cells [61]. Expression of a miR824-resistant *AGL16* mRNA, but not the wild-type *AGL16* mRNA induced, excessive satellite meristemoid lineages. In contrast, plants overexpressing *miR824* decreased the incidence of high-order stomatal complexes. *miR824* was supposed to be able to transport through PD [107]. Indeed, several screens for mutants defective in stomata development have identified some genes affecting PD transport. *miR399* acts as a long-distance signal, it negatively regulates the ubiquitin-conjugating E2 enzyme PHO2 and contributes to the regulation of stomatal development and phosphate homeostasis [108]. *SPCH*, *EPF2*, and *TMM* transcripts are up-regulated in *35S::MIR399b* plants. The SPCH protein is restricted to MMCs and meristemoids but altered plasmodesmatal permeability led to ectopic location of AtSPCH [109]. *chorus* is a weak recessive allele of *GSL8*, which encodes a callose synthase [110]. SPCH-YFP readily diffuses from cell to cell via PD in the *chorus* mutant [24]. Stomatal clusters were formed in this mutant due to the excessive range of SPCH movement caused by increased plasmodesmal permeability in the mutants. KOBITO1 is a putative glycosyltransferase-like protein previously implicated in cellulose biosynthesis, the *kob1-3* mutation leads to the formation of stomata clusters in the *erl1 erl2* background, but it is normal in the wild type background [111]. However, the other mutants defective in cellulose biosynthesis do not show *kobito1-like* phenotypes [112], suggesting KOBITO1 may function in another, as yet unknown pathway restricting PD movement. These findings support the idea that callose-mediated restriction of PD permeability is important to stomatal patterning (Figure 1).

**Figure 2:**
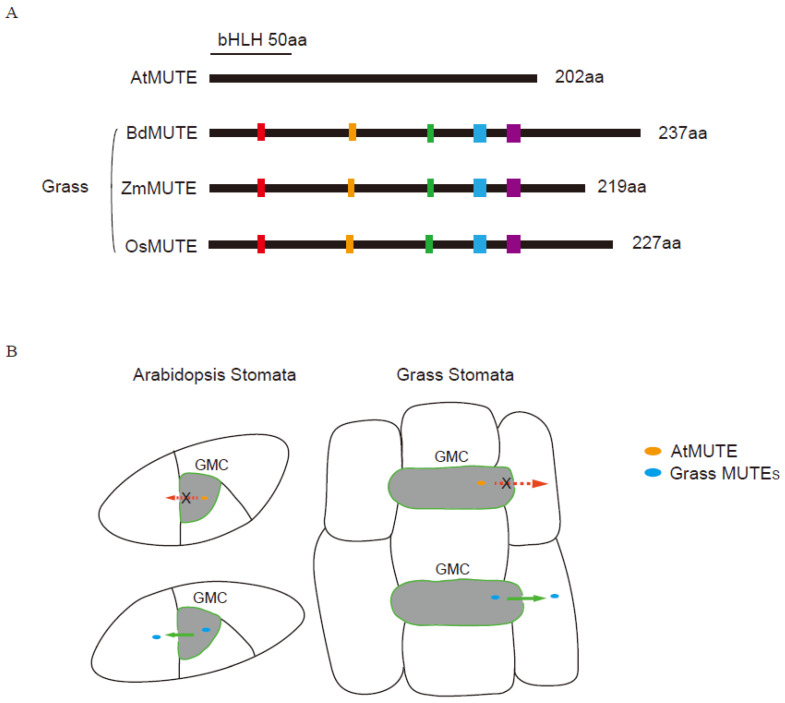
Schematic diagrams showing the mobility of homologous MUTE proteins in *Arabidopsis* and grasses. (**A**) Schematic diagram of potential residues controlling the mobility of MUTE proteins. MUTE orthologs of the representative grass species *Brachypodium* (BdMUTE–Bradi1g18400), maize (ZmMUTE-GRMZM2G417164) and rice (OsMUTE, LOC_Os05g51820) were aligned with the representative eudicot species *Arabidopsis* (AtMUTE, At3g06120). Colored-boxes represent the potential motifs responsible for mobility regulation. (**B**) Mobility of MUTE orthologues in grass and *Arabidopsis*. In *Arabidopsis*, *YFP-BdMUTE/ZmMUTE/OsMUTE* driven by the GMC-specific *AtMUTE* promoter was visible in the neighboring cells, unlike GMC-only expression of *AtMUTE-YFP* (Left panel). *AtMUTE-YFP* expressed in grass stomata driven by the promoter of grass *MUTE* is restricted in GMC and does not move to the neighboring cells, whereas BdMUTE, ZmMUTE, and OsMUTE can move from the GMC to the adjacent SMC (Right panel). GMC (guard mother cell), dark gray. Grass MUTE proteins, blue oval. AtMUTE protein, orange oval. Red/Green arrows indicate the MUTE proteins cannot/can move through plasmodesmata, respectively.

*BdMUTE* encodes a bHLH transcription factor associated with GMC identity, which is an ortholog of the stomatal regulator *AtMUTE* [58]. The specification of lateral SCs by BdMUTE appears due to its acquisition of cell-to-cell mobility from GMCs to SMCs [58] (Figure 2). This behavior is similar to that of ZmMUTE/BZU2, which is required for not only SMC differentiation but also GC division [60]. When expressed in rice, the fluorescence of YFP-BdMUTE, YFP-ZmMUTE and YFP-OsMUTE driven by GMC-specific promoter of *OsMUTE* were first detected in the early GMCs, then appeared in the SMCs, indicating their similar mobility [60]. In addition, both YFP-BdMUTE and YFP-ZmMUTE expressed in *Arabidopsis* with the GMC-specific *AtMUTE* promoter were visible in the neighbor cells of GMC [60] (Figure 2). In contrast, the OsMUTEp:YFP-AtMUTE expressed in rice was not detected in SMCs, and the pBdMUTE::AtMUTE-YFP fluorescence was restricted to GMCs and GCs in *Brachypodium*, a similar behavior like that of AtMUTE in *Arabidopsis* [58,60] (Figure 2). In addition, the pBdMUTE::AtMUTE-YFP failed to rescue the SC and GMC defects in the *BdMUTE* loss-of-function mutant [58].These results imply the potential divergent characteristic and function of MUTE proteins in different species (Figure 2).

MUTEs are small proteins, either those symplastic connections allow for its passive transport, or certain domains within BdMUTE define its mobility (Figure 2). MUTE proteins from different species have a variable C-terminal region, whereas the N-terminal region is relatively conserved [60]. Wang et al. generated truncated MUTE proteins lacking 30 amino acids in C-terminal (indicated byΔC) to investigate their mobility. The fluorescence of AtMUTEp:YFP-ZmMUTE-ΔC and AtMUTEp:YFP-BdMUTE-ΔC were only detected in the GMCs, which is similar to the pattern of AtMUTEp:AtMUTE-YFP. YFP-ZmMUTE-ΔC and YFP-BdMUTE-ΔC are only located in the early GMCs, and not in the SMCs and young SCs when expressed by *OsMUTE* promoter in rice [60]. Based on these assays, 30 amino acids of the C-terminus were thought to be necessary for the mobility of ZmMUTE and BdMUTE.

The GRAS transcription factor SCARECROW (SCR) and SHORTROOT(SHR) are notable regulators involved in multiple development processes in plants [113]. The SHR/SCR module functions in endodermis development of *Arabidopsis* and maize roots, but regulate different cell type in leaf between *Arabidopsis* and maize [114]. The ectopic expression of *ZmSHR1* in rice bundle sheath cells resulted in ultra-stomatal rows between veins (Figure 1). It was supposed that SHR might act as a mobile signal originated from vein precursor cells and executed together SCR to establish the GC lineage [54,115], and *OsSCR*/*OsSHR* thus regulates the initiation of stomatal lineage cells and the formation of SCs.

The double mutant *osshr1 osshr2* showed abnormal stomata. Stomata with anomalous SCs and stomatal density decreased in *osscr1* mutant; however, mutation of *OsSCR2* showed a normal stomatal phenotype [59]. In addition, OsSPCH and OsMUTE might activate *OsSCR* by directly binding to its promoter. This suggests that the grass SHR/SCR module not only controls vein development in maize [54,115,116], but also participates in the development of stomata complex in rice [54].The permeability of PD is regulated not only by callose but also their architecture. PLM encodes a putative enzyme involved in the biosynthesis of sphingolipids with very-long-chain fatty acid. Loss of PLM enhances plasmodesmatal permeability by altering the inner structure of cytoplasmic sleeve in PD [117]. Arabinogalactan proteins (AGPs) are a gene family of extracellular proteoglycans. Hyp O-galactosyltransferase (HPGT) encodes a critical enzyme that functions in AGP biosynthesis in *Arabidopsis*. The reduction in the formation of functional AGPs in *hpgt1,2,3* triple mutant led to two or more stomata clusters. This phenomenon is ascribed to the change of PD structure with increased highly permeable complex branched PD, which may increase PD permeability [118].

## 5. PD Distribution at Different Cell-to-Cell Interfaces

Guard cell opening and closing are engaged by ions (K^+^, Cl^−^) that function as a rapid response signal [119,120], the pumping or exclusion of ions changes the osmotic potential, resulting in changing the turgor pressure. Thus, stomata should be rather sensitive and require a safe and controllable connection with surrounding cells. Usually, the mature guard cells are not connected with neighboring epidermal cells by functional plasmodesmata in most seed plants, this situation may be able to maintain their different osmotic potential and may impel the ions traffic intercellularly in a transmembrane manner. However, plasmodesmata were observed in ventral walls between sister guard cells and, most importantly, between GCs and epidermal cells in both *Vicia faba*, *Allium cepa*, *Beta vulgaris* diand tobacco [41,121]. Microinjection experiments indicated that fluorescent dye injected into the GMCs and immature GCs of *A. cepa* and *Commelina communis* L. spread rapidly into surrounding cells, but no diffusion happened when injected into mature GCs or SCs [79]. However, one exception is that fluorescent dyes can move from one guard cell to another in fern stomata [122], which is consistent with the observation of PD between two GCs [119]. This symplastic connection between guard cells in fern suggests a potential distinct regulation mechanism for stomatal movement. Even so, hyperpolarizing pulses applied to one guard cell will not provoke Ca^2+^ signal transport to another, suggesting the electrical conductance of the plasmodesmata between stomata of fern is limited [122]. One more case is that PD are present between ordinary epidermal cells and SCs, but not between GCs and SCs and two GCs in maize, but the fluorescent dye BCECF [(20,70-bis-(2-carboxyethyl)-5(and6) carboxyflurescein] can move between two GC of maize unexpectedly in an unknown way [123]. Thus, SCs are not isolated but connected to adjacent epidermal cells, which is different from GCs. Overall, the present of functional plasmodesma within the stomatal complex may differ somewhat between different cell types and plant species.

## 6. Organelle-Nucleus-Plasmodesmata Signaling (ONPS) and Stomata Movement

PD allows cell-to-cell movement of various metabolites and signaling molecules and therefore play critical roles in responding to environmental signals and stresses. In addition to sugars and mobile proteins, hormones can move from cell to cell through PD. Gibberellic acid (GA) and ABA were found to be transported via PD in *Chara vulgaris* and *P. patens*, respectively, and the diffusion of auxin via PD is essential for the hypocotyl phototropism and lateral root development [124,125,126]. Furthermore, the level of plasmodesmatal callose is regulated by hormones. ABA regulate the expression of PD proteins involved in callose synthesis/ degradation, for instance CalSs, PLASMODESMATA-LOCALIZED PROTEINS (PDLPs) and β-1, 3 glucanases to modulate PD permeability and molecular trafficking [127]. Signaling originating from other organelles (such as the chloroplasts and mitochondria) functions in influencing PD formation and function. These signals act in the nucleus to alter the expression of genetic pathways that control both plasmodesmatal permeability and density. This pathway was named ONPS. SAL1(nucleotide phosphatase)-PAP(3′-phosphoadenosine-5′-phosphate) retrograde pathway serves as a second messenger and interacts with ABA signaling to regulate stomatal closure in *Arabidopsis* [128]. It is thus possible that PD participates in this process, but more direct evidence is required to support this speculation.

## 7. Perspectives

Stomata are essential for plant development; mutations blocking stomatal formation are lethal [48,51,57,129]. Striking differences in stomatal patterning are noted in comparisons of monocot and eudicot leaves. Namely, stomata appear to be randomly distributed across the epidermis of eudicot leaves, whereas monocot stomata are arranged in evenly distributed, parallel rows [13,14,15]. At the proximal base of the growing leaf blade, stomatal precursor cells arise in cell files with higher rates of cell division relative to flanking cell files. Importantly, this proliferation phase is much more abbreviated compared with that in *Arabidopsis*, and another major distinction is that grasses lack a self-renewing meristemoid phase. Every cell in the precursor cell file undergoes one asymmetric division in the same orientation (i.e., parallel to the marginal axis of leaves) to produce a smaller GMC and a larger inter-stomatal sister cell. In grasses, cells in the flanking files become polarized in response to cues from the GMC and divide asymmetrically to become SMCs [130]. During these different patterning stages of stomatal development in dicot and monocot, both conserved and distinct regulation mechanisms are uncovered. For example, the MUTE and FAMA transcription factors play important roles in both dicot and grasses, while *PAN1* and *PAN2* only function in SC formation in monocots. Notably, ZmMUTE is involved in the regulation of both GC and SC development in maize [60]. Whether other mobile factors, except MUTE, are involved in the SC formation is still elusive. Moreover, whether the PD allow bi-directional movement between GMCs and SCs is also unknown. It is, thus, possible that there are also feedback signals from SMCs to GMCs, which need further investigation.

Symplastic trafficking between neighboring cells mediated by PD is one of the efficient ways for the short-distance transport of mobile molecules to function non-anonymously. Based on the present studies in stomatal development, it is speculated that the GC differentiation, either in dicots or monocots, requires a relative isolated cellular environment with blocked symplastic communication between GC and neighboring epidermal cells. This may on the one hand protect GCs from disturbing by exterior signals, and prevent the specific factors function at specific stages trafficking outside. However, this situation changes when SCs in grasses start differentiation, which need signals transported from GCs to activate the following steps. Symplastic communication between these two cell layers is required. Thus, the changes of symplastic trafficking and PD permeability are dynamically dependent on the stomata developmental stages. The underlying regulation mechanism remains unclear to date. Studies on more plant species would provide more clues.

## Figures and Tables

**Table 1:** Experimental verified mobile factors involved in stomatal development in grasses and *Arabidopsis*.

Gene Name	Species	Movement Track	Gene Function	References
*ZmMUTE*	*Z. mays*	GMCs to non-stomatal neighbor cells	Recruitment of SCs, GMC identity and SMC polarization	[60]
*OsMUTE*	*O. sativa*	GMCs to non-stomatal neighbor cells	Recruitment of SCs, GMC identity and SMC polarization	[59]
*BdMUTE*	*B. distachyon*	GMCs to non-stomatal neighbor cells	Recruitment of SCs, GMC identity and SMC polarization	[58]
*ZmSHR1*	*Z. mays*	BSC to stomatal lineage cells	Promotes stomatal initiation and SCs formation	[54]
*OsSHR1*	*O. sativa*	BSC to stomatal lineage cells	Promotes stomatal initiation and SCs formation	[59]
*OsSHR2*	*O. sativa*	BSC to stomatal lineage cells	Promotes stomatal initiation and SCs formation	[59]
*miR824*	*A. thaliana*	SLGC to GC	Regulates satellite meristemoid division	[107]

GCs, guard cells; GMC, guard mother cell; SMC, subsidiary mother cell; SCs, subsidiary cells; BSC, bundle sheath cells; SLGC, stomatal lineage ground cell.
